# Development and evaluation of an IoT-based hypertension surveillance system in community health settings: a mixed-methods quasi-experimental study protocol

**DOI:** 10.1186/s12913-025-13562-3

**Published:** 2025-10-22

**Authors:** Wen Zheng, Li-li Hua, Jie Tan, Jing-shi Zhang, Yan Liang, Jing Pan, Ming-xia Yu, Jing-wen Gan

**Affiliations:** Liyuan Community Health Service Center, Tongzhou District, Beijing, China

**Keywords:** Hypertension, IoT technology, Primary healthcare, Blood pressure control, Cost-effectiveness, Implementation process

## Abstract

**Background:**

Hypertension, affecting over 1.3 billion people globally, poses a significant public health challenge. Despite its widespread impact, hypertension management in primary healthcare settings faces challenges such as fragmented data collection, low patient adherence, and insufficient real-time monitoring. This study evaluates the effectiveness, cost-effectiveness, and implementation process of an Internet of Things (IoT)-based hypertension surveillance system designed to address these gaps in community health settings.

**Methods:**

This pragmatic quasi-experimental trial involves 2,000 hypertensive patients managed under family doctor contract services in Beijing, China. The intervention group (*n* = 1,000) utilizes an IoT-based hypertension monitoring platform, which integrates smart blood pressure devices and real-time data transmission for dynamic tracking and management. The control group (*n* = 1,000) receives standard care without IoT integration. The primary outcome is blood pressure control rate, while secondary outcomes encompass standardized management rate, measurement completion rate, referral rates and biomarker profiles. Cost-effectiveness is evaluated using administrative data on healthcare utilization and intervention costs. Qualitative data, collected through patient surveys and focus groups with healthcare providers, explores implementation barriers and user experiences.

**Discussion:**

The study aims to provide comprehensive evidence on the clinical effectiveness, cost-effectiveness, and implementation process of IoT-based hypertension management in primary care. By combining quantitative and qualitative methods, it seeks to understand how the intervention works in real-world settings and identify facilitators and barriers to its implementation. The findings could inform policy decisions and optimize resource allocation for chronic disease management in primary care.

**Trial registration:**

ChiCTR, ChiCTR2500103556.Registered 30 May 2025.

**Supplementary Information:**

The online version contains supplementary material available at 10.1186/s12913-025-13562-3.

## Background

With the accelerating global aging process, the prevalence of chronic diseases, particularly hypertension, which is a leading cause of cardiovascular diseases and stroke, has been steadily increasing, posing significant challenges to public health worldwide, affecting over 1.3 billion people worldwide [1]. According to the China Cardiovascular Disease Report 2018, 27.9% of Chinese residents aged 18 and above have hypertension. The prevalence rate of hypertension increases significantly with age. Specifically, more than 50% of those aged 65 and above are hypertensive [2]. In China, the treatment rate of hypertension among residents aged 18 and above is 45.8%, and the control rate is 16.8%. The overall prevention and treatment situation of hypertension still requires further improvement [3]. In 2013, the total health expenditure in China amounted to $462.74 billion, among which the direct economic burden of hypertension accounted for 6.6% [2]. It is estimated that the standardized community management of hypertension can save approximately $30.45 in annual per capita direct medical expenses for patients with hypertension. Moreover, an annual per capita investment of $116 in the community health management of hypertension in China can generate a positive net benefit, that is, the output exceeds the input [4]. Effective management of hypertension is not only critical for individual health but also has profound implications for the allocation and utilization of healthcare resources.

Despite widespread attention to hypertension prevention and control globally, the management outcomes remain suboptimal [5, 6]. Particularly in primary healthcare settings, hypertension management faces numerous challenges. the quality of hypertension management in primary healthcare institutions varies significantly, with notable disparities between urban and rural areas. Urban regions, with relatively abundant medical resources, tend to have more standardized hypertension management practices, whereas rural areas, due to limited healthcare resources, often struggle with inadequate management. Meanwhile, general practitioners in primary healthcare settings often lack specialized training and guidance in hypertension management, resulting in insufficient standardization and scientific rigor in their practices. In addition, some patients have an insufficient understanding of the hazards of hypertension. In rural areas, patients are often reluctant to take prescribed medications if they perceive no symptoms. Moreover, there is a shortage of general practitioners, and patients have a weak awareness of self-management, which can easily lead to the interruption of follow-ups [7]. All in all. community health centers play a critical role in hypertension management, but traditional approaches often face challenges such as fragmented data collection, lack of real-time monitoring, and low patient adherence. The integration of Internet of Things (IoT) technology into hypertension management offers a promising solution [8].

With the rapid development of the IoT technology, its application in healthcare has become increasingly feasible. IoT technology enables real-time monitoring of patients’ physiological data through sensors and smart devices, transmitting this data to cloud platforms for analysis and processing, thereby providing timely health management recommendations to both physicians and patients [9]. In Japan, the Internet of Things (IoT) is applied through the CureApp HT digital therapeutics app. A home blood pressure monitor, as an IoT device, is used by patients to measure their blood pressure daily. A total of 390 patients with essential hypertension aged 65 years or younger who were not taking antihypertensive medications were divided into two groups. The group using CureApp HT in addition to lifestyle modification guidance showed a significantly greater reduction in blood pressure compared to the control group. Moreover, in terms of cost-effectiveness, the incremental cost-effectiveness ratio of using the app under a specific model is lower than the threshold in Japan, suggesting it may be cost-effective over a lifetime [10]. In Italy, the Tholomeus^®^ web is widely used. which runs in the context of the Internet-of-Medical-Things (IoMT), with operations spanning various healthcare and home settings. It serves as a crucial telehealth platform for chronic disease management, especially hypertension. For instance, in general practices, the telehealth solution has helped family doctors better manage hypertensive patients. In hospitals, it has enabled the identification of different hypertension types and provided insights into patients’ vascular health. The home monitoring has also increased patients’ awareness of their health status, facilitating timely medical advice and potentially improving long-term health outcomes [11].

IoT-based hypertension management information systems are currently lacking in China, despite the technology’s encouraging potential in this area, particularly in primary healthcare settings [12]. Most existing hypertension management systems rely on traditional manual records and paper-based archives, making it difficult to ensure the timeliness and accuracy of data. Furthermore, these systems often lack effective integration with family physician contracting services, hindering dynamic monitoring and performance evaluation of hypertension management. Therefore, the development of an IoT-based hypertension management information system is essential to enhance the scientific rigor and standardization of hypertension management while providing primary healthcare institutions with effective tools for performance evaluation. Through devices such as smart blood pressure monitors and health huts, patients’ blood pressure data can be uploaded in real time to health management platforms, enabling physicians to monitor blood pressure trends and adjust treatment plans accordingly. Simultaneously, the system can provide real-time monitoring data for managers to evaluate the performance of family physician contracting services, thereby improving the quality of hypertension management in primary healthcare settings.

### Objectives

The general aim of this study is to evaluate the effectiveness, cost-effectiveness, and implementation process of an IoT-based hypertension surveillance system in primary healthcare settings in China. Specifically, the study addresses the following research questions:

1. Clinical Effectiveness: ①What are the effects of the IoT-based monitoring system on blood pressure control rates and standardized management rates compared to traditional care?②Does the intervention improve secondary outcomes, including referral rates for refractory hypertension and biomarker profiles (e.g., lipids, glucose)?
2. Economic Evaluation: What are the incremental costs and cost-effectiveness of the IoT system versus standard care?
3. Implementation Process: What are the facilitators and barriers to deploying the IoT system in routine primary care, as perceived by healthcare providers and patients?

## Methods

### Overall study design and setting

**Fig. 1:**
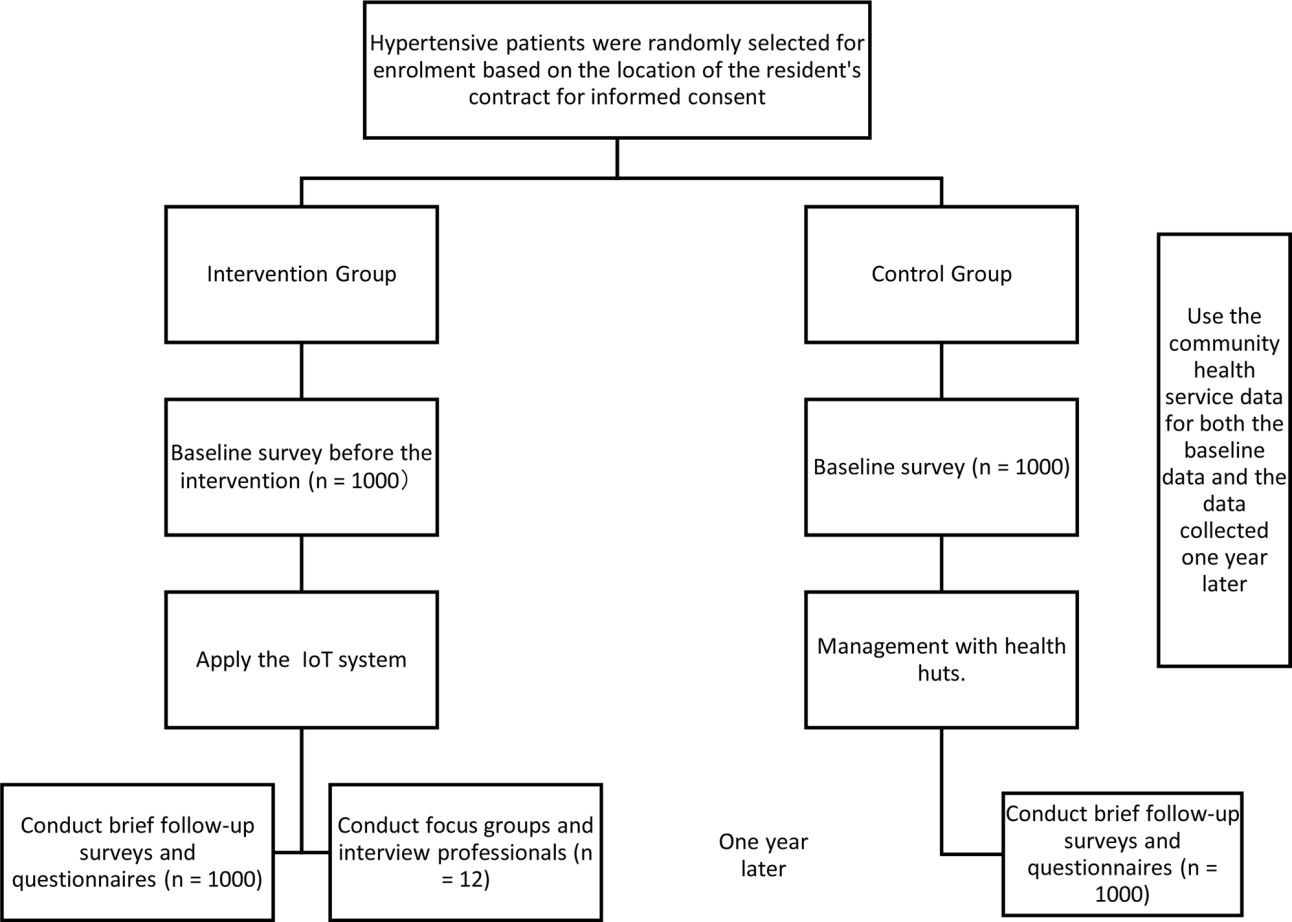
Flow diagram of the study design

**Fig. 2:**
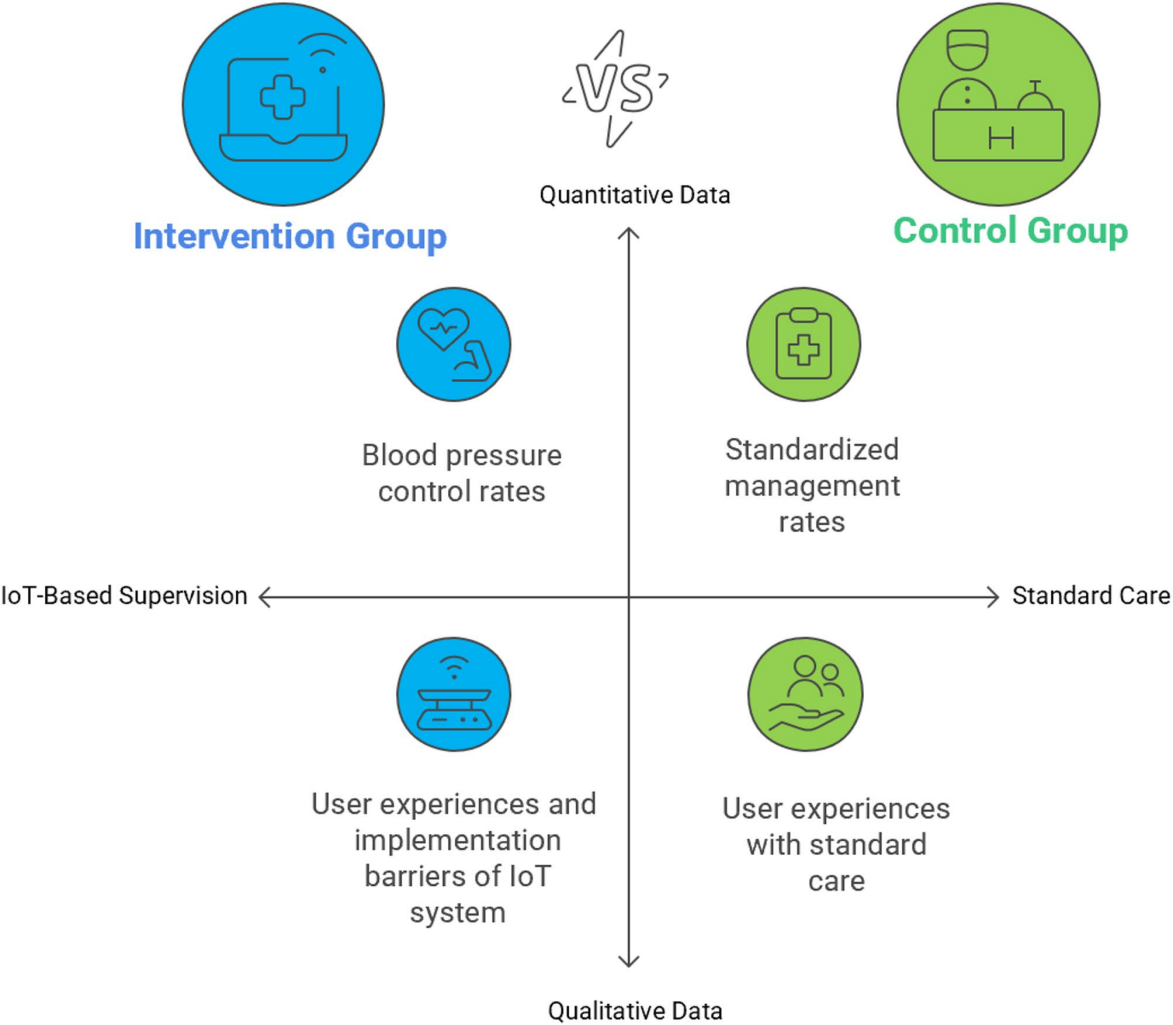
Comparative analysis of hypertension management strategies

This study is a pragmatic quasi-experimental trial aimed at evaluating the effectiveness and feasibility of an IoT-based hypertension monitoring and management system in primary care settings. The overall study design is described in Fig. 1. The study compares outcomes between an intervention group (IG) using the IoT system and a control group (CG) receiving standard care. Quantitative data will assess clinical outcomes including blood pressure control rates, standardized management rates, and healthcare utilization, while qualitative methods will examine user experiences and implementation barriers through surveys and interviews with patients and healthcare providers (Fig. 2).

The study is carried out in Liyuan Health Center and its affiliated community health service stations in Tongzhou District, Beijing, P.R. China. The research area covers a large population of hypertension patients. It serves a population of approximately 250,000 people in urban and suburban regions, where the hypertension prevalence reaches as high as 33.3% [3]. The intervention is deployed across five family doctor teams in Liyuan Community Health Center and Xiaogao Community Health Station. These sites represent typical primary care institutions in Beijing’s urban expansion areas, serving mixed populations of long-term residents and recent migrants. The center utilizes Beijing’s municipal health information platform (B/S architecture) and has pre-existing health huts with basic measurement capabilities. The new IoT system integrates with these platforms through application program interfaces (APls) [13], enabling real-time data transmission from 6 intelligent monitors to electronic health records.

During the clinical practice application stage, all research subjects are randomly selected from the currently managed patients. 1000 hypertension patients who have signed contracts with the family doctor teams of 5 teams in Liyuan Health Center and Xiaogao Community Health Service Station where the system is deployed are selected as the intervention group. These patients use the IoT-based hypertension supervision platform for blood pressure measurement, and their data is included in the new system for statistical analysis. Another 1000 hypertension patients who have signed contracts with the family doctor teams of other subordinate stations in Liyuan Health Center where the system is not deployed are selected as the control group. They only measure blood pressure in the health huts, and their data is not included in the new system for statistical analysis.

### Study intervention

**Fig. 3:**
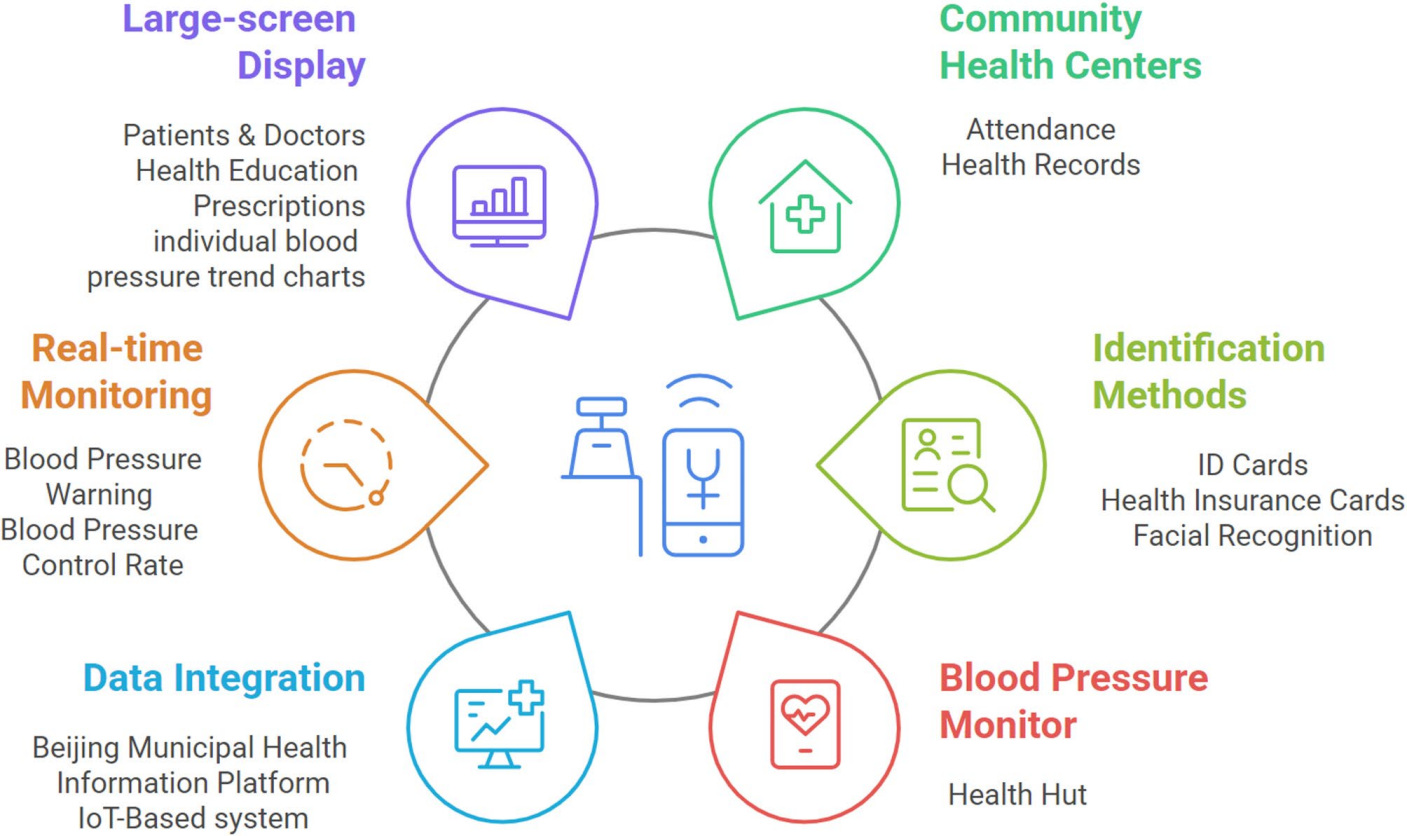
IoT-based hypertension surveillance system

The study intervention focuses on the development and application of IoT-based hypertension monitoring and management information system. This system integrates intelligent blood pressure monitoring devices and software to dynamically track and manage hypertensive patients in primary care settings. The intervention involves deploying six blood pressure health management all-in-one machines, two electronic display screens, and an intelligent hypertension monitoring information system in the general practice clinics of Liyuan Community Health Center and Xiaogao Community Health Station. These devices are connected to the health hut and the residents’ personal health records, enabling real-time data transmission and analysis. The system utilizes identity information, health insurance cards, and facial recognition to ensure accurate data collection and upload. The primary goal is to monitor blood pressure data, control rates, and standardized management rates. By leveraging IoT and AI technologies, the intervention seeks to enhance the efficiency and quality of hypertension management in primary care (Fig. 3).

The system’s innovative features include interoperability with existing health hut, automated risk assessments, and intelligent decision support. It employs a modular design for scalability, enabling future expansion to additional metrics like blood glucose and weight, while standardized data formats and open interfaces facilitate integration with external platforms such as the Beijing Community Health’s electronic health record (EHR) and Tongzhou District Performance Evaluation Platform. Security is ensured through role-based access control [14]. Key functionalities include real-time data transmission (via ID card, medical insurance card, or facial recognition), dynamic tracking of standardized management and control rates, and dashboard visualization of blood pressure trends for clinicians and patients. During development, rigorous testing was conducted: 500 simulated patient datasets were used to validate system functionality, including seamless data upload to individual health records and public health systems, accurate calculation and display of monthly/quarterly metrics on clinic screens, automated risk alerts for uncontrolled hypertension, and generation of quarterly/annual trend reports. The system successfully passed testing, confirming capabilities in risk alerts, statistical analysis, and user interface usability, and is now operational in the clinical application phase. In the clinical phase, 2000 hypertensive patients are chosen at random from the current management pool to participate in the trial. The intervention group (*n* = 1,000) consists of patients from five family doctor teams in Liyuan Community Health Center and Xiaogao Station equipped with the new system, while the control group (*n* = 1,000) comprises patients from other stations without the system. The intervention group uses the IoT-based system for blood pressure measurements, with data automatically uploaded to health records and displayed on screens.

Patients in the intervention group can view their daily blood pressure conditions on the display. If there are any abnormalities, they can receive timely treatment. They can also print out the results, health education prescriptions, and blood pressure trend charts on-site. Managers can inquire about the number of people taking blood pressure measurements, the standardized management rate and the blood pressure control rate of each team in our hospital on a daily, monthly and quarterly basis. Once a month, the information is summarized, a team meeting is held to analyze the data collected by the system, statistics are analyzed and made public, the team is urged to strengthen the blood pressure management and strive to improve their own level, and the doctors are urged to refer patients affecting the control rate to the higher-level hospitals so as to form a good diagnosis and treatment pattern. The control group is managed in the traditional way. Patients only have their blood pressure measured in the health hut, and the data is not included in the new system for statistics. Management is carried out in accordance with the requirements of the “National Basic Public Health Service Specification (Third Edition) [15]”.

### Sample size calculation

A formal power calculation was performed a priori using G*Power software (version 3.1.9.7) for the primary outcome: the between-group difference in blood pressure control rate.

The anticipated control rate in the control group (P₁) was conservatively estimated at 45%, informed by historical data from our health center and findings from literature on usual care in similar settings [16]. We hypothesized that the IoT-based intervention would yield a clinically meaningful absolute improvement of 10% points, raising the control rate in the intervention group (P₂) to 55%. This effect size is supported by a recent meta-analysis of mHealth interventions [16], which reported a significant absolute improvement yet considerable heterogeneity among studies.

Using a two-sided significance level (α) of 0.05 and a power (1-β) of 80%, the calculation indicated a minimum requirement of 350 participants per group (total *n* = 700). Accounting for an estimated 15% attrition rate, the sample size was adjusted to 412 per group (total *n* = 824).

However, considering that there are more than 80,000 hypertension patients in this region and approximately 20,000 hypertension patients who have signed contracts with family doctors in this community, in order to achieve better research results and based on the available research resources, it was finally decided to include 2,000 patients, with 1,000 patients in the intervention group and 1,000 patients in the control group. This larger sample size ensures robust power for the primary and all secondary outcomes, enables precise subgroup analyses, and provides high-quality, generalizable evidence essential for informing public health policy on chronic disease management.

### Participants, selection criteria and recruitment

The study targets hypertensive patients managed under the family doctor contract service at Liyuan Community Health Center and its subordinate Community Health Station in Tongzhou District, Beijing. The intervention group (IG) will consist of 1,000 hypertensive patients randomly selected from five family doctor teams at Liyuan Community Health Center and subordinate Xiaogao Station where the IoT-based hypertension monitoring system is deployed. The control group (CG) will include another 1,000 hypertensive patients from other stations without the system, matched for baseline characteristics.

Participants must meet the following inclusion criteria to be enrolled in the study. First, they must be aged 35 years or older and have a confirmed diagnosis of primary hypertension as defined by the Chinese Hypertension Clinical Practice Guidelines [17], which requires systolic blood pressure ≥ 140 mmHg and/or diastolic blood pressure ≥ 90 mmHg measured on at least two separate occasions to ensure diagnostic accuracy. Second, they must be residents within the administrative jurisdiction of Liyuan Health Center and have signed a family doctor contract, which is a prerequisite for accessing hypertension management services under the National Basic Public Health Service Program [18]. This ensures that participants are eligible for the standardized care and monitoring provided by the health center’s healthcare teams. Third, participants must demonstrate the functional capacity to attend outpatient visits either independently or with minimal assistance, excluding those who are bedridden or require full-time care. This criterion is essential to ensure that participants can engage in regular blood pressure measurements, follow-up consultations, and health assessments as required by the study protocol, thereby facilitating the collection of reliable data on hypertension management outcomes.

Participants will be excluded if they meet any of the following criteria to ensure the safety and validity of the research results. First, individuals with a history of acute cardiovascular or cerebrovascular events, such as stroke, myocardial infarction, or aortic disease, occurring within the past 12 months are excluded, as these conditions may confound the assessment of hypertension management outcomes and require specialized clinical interventions that could interfere with the study’s standardized procedures. Second, participants with severe comorbidities, including heart failure, severe renal failure, or cancer, are ineligible, as their complex medical conditions may reduce their capacity to adhere to the study’s monitoring protocols and could lead to substantial variability in health-related outcomes. Third, individuals with uncontrolled psychiatric conditions (such as schizophrenia or severe depression with suicidal ideation) will be excluded, as these conditions may compromise study participation, data accuracy, or patient safety. Fourth, patients aged over 80 years or those with severe mobility impairment (defined as the inability to ambulate even with assistive devices) are excluded, as their limited physical capacity may hinder regular attendance and adherence to the study’s follow-up schedule. Finally, pregnant or lactating females are excluded due to the unique physiological changes and medication considerations during this period, which require specialized care outside the scope of the study’s focus on primary hypertension management in the general adult population. These exclusion criteria are designed to minimize confounding variables and ensure that the results reflect the true impact of the intelligent monitoring system on hypertension patients under routine primary care settings.

Eligible participants will be identified through Liyuan Health Center’s electronic health record (EHR) system, which maintains a registry of approximately 20,000 registered hypertension patients. The research team will use EHR data to pre-screen candidates, filtering for age, diagnosis, contract status, and exclusion criteria, after which random selection will be performed using a computer-generated random number table, stratified by age and sex to ensure demographic balance. Meanwhile, the control group (CG) will match to the intervention group (IG) on age, sex, and hypertension duration using EHR data to minimize baseline confounding.

### Study conduct

Eligible patients will be recruited via text messages or in-person during clinic appointments, explaining the study objectives, procedures, and voluntary nature of participation. Those interested will be contacted by research nurses for a telephone or in-person consultation to review the study details, including risks, benefits, and data confidentiality. Written informed consent will be obtained prior to enrollment. Upon consent, participants will complete a baseline assessment comprising: (1) demographics (age, sex, education); (2) clinical metrics: blood pressure (measured twice at rest, 5 min apart), BMI, fasting labs for lipids/glucose/renal/liver function, duration of hypertension, known comorbidities (e.g., diabetes, coronary artery disease); (3) functional assessment: mobility status (e.g., walking ability, use of assistive devices) and adherence to hypertension management (e.g., medication compliance, clinic visit frequency).

Following blood pressure measurement, the intervention group’s data will be automatically transmitted to the electronic health record system via IoT-enabled devices, where values are instantly integrated into statistical analyses and visualized on clinic display screens for real-time monitoring by both clinicians and patients. The system will trigger alerts for uncontrolled hypertension (systolic blood pressure is greater than 180 mmHg or diastolic blood pressure is greater than 110 mmHg [17]), enabling timely clinical decisions (medication adjustments/referrals). Participants will receive printed personalized reports with trend analyses and educational materials after each measurement. The control group will receive standard care via traditional health huts without IoT integration, with data manually recorded in electronic health records.

A three-month interval is deemed adequate for the initial follow-up, given the service intensity and participants’ functional capacities. Both groups will undergo quarterly follow-ups (three months, six months, nine months, one year) to collect the primary outcome (blood pressure control rate) and secondary outcomes (standardized management rate, measurement completion rate, referral rates for refractory hypertension, changes in biochemical indicators like lipids and fasting glucose), with data collected from IG’s real-time IoT measurements and CG’s traditional health hut’s records. If participants do not proceed with subsequent follow-ups, they will be contacted by phone and encouraged to continue their involvement.

At the conclusion of the one-year trial, questionnaire surveys and interviews with professionals will be carried out. Professional interviews will be conducted via focus groups at the trial’s end. The discussions will be structured around three key themes: (1) System usage experiences: Exploring practical aspects of the IoT-based hypertension surveillance system, including its integration into clinical workflows, data accuracy, and any adaptations made during deployment. (2) Implementation challenges and facilitators: Identifying organizational factors (e.g., resource allocation), technical factors (e.g., device connectivity), and patient-related factors (e.g., adherence behaviors) that influence system effectiveness. (3) Program theory refinement: Examining how the intervention’s components (real-time monitoring, alerts, patient engagement tools) interact to achieve outcomes, and proposing improvements to optimize hypertension management pathways. Sessions will include physicians, nurses, and administrators to capture diverse perspectives. Additionally, assistance is provided throughout the study to help complete the survey, particularly for individuals with visual impairments or other difficulties.

### Outcomes and variables

The primary outcome of the trial is blood pressure control rate, which is the proportion of patients whose blood pressure is maintained within the target range. According to the National Basic Public Health Service Specification (Third Edition) [15], blood pressure should be controlled below 140/90 mmHg for individuals aged 35–65 years, and below 150/90 mmHg for those aged 65 years and above. Secondary outcomes: (1) standardized management rate: Calculated as the proportion of patients in the intervention and control groups who receive management in accordance with the National Basic Public Health Service Specification (Third Edition) [15], to evaluate the impact of the system on the standardization of hypertension management; (2) blood pressure measurement completion rate: The number of blood pressure measurements completed per patient compared to the expected quarterly measurements; (3) Referral rates: The number of patients referred to higher-level hospitals due to refractory or secondary hypertension, and the proportion of referred patients whose blood pressure stabilizes upon return; (4) Biochemical indicators: Changes in lipid profiles and other relevant biomarkers.

In addition, exploratory outcomes are economic evaluation(cost-effectiveness: analysis of system deployment costs versus improvements in hypertension control and reduced referrals; performance incentives: correlation between system-generated data and family doctor contract fulfillment metrics for incentive allocation), patient satisfaction (the subjective experiences and perceived effects), implementation barriers (engage professionals in focus groups and interviews to examine implementation barriers and improvement opportunities from the service providers’ perspective).

**Fig. 4:**
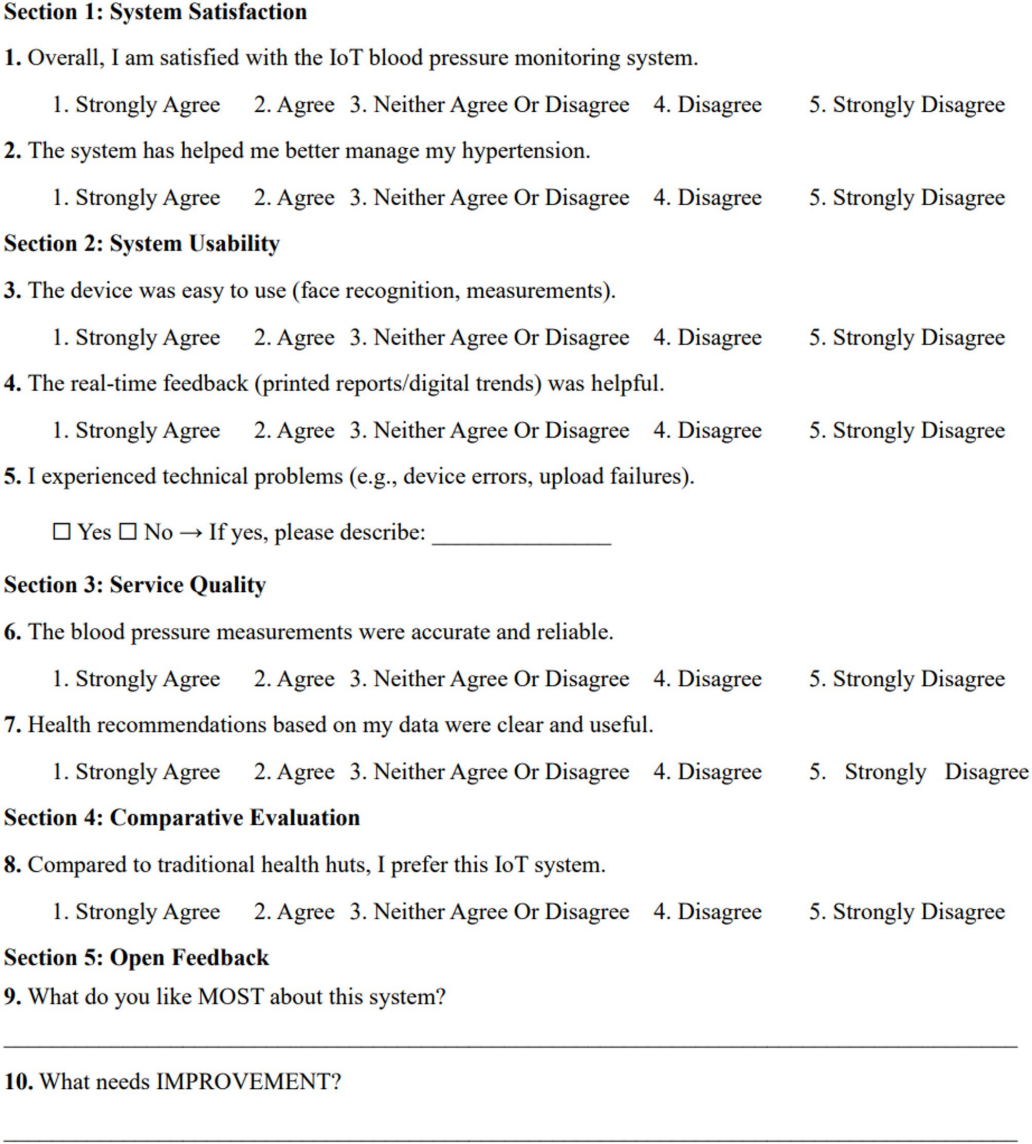
IoT-based hypertension management system satisfaction questionnaire

The satisfaction of patients in the control group is evaluated through a questionnaire survey with three questions:1. How satisfied are you with the overall hypertension management services currently provided by the community health service center? 2. How satisfied are you with the following aspects of the service? (a) Convenience of blood pressure monitoring. (b) Professional guidance from family doctors. (c) Timeliness of follow-up visits. A five-point Likert scale is used with the following response options: Very dissatisfied, somewhat dissatisfied, neutral, somewhat satisfied, very satisfied. 3. What aspects of the current hypertension management service do you think need the most improvement? (Open-ended question). Satisfaction in the intervention group is assessed using a dedicated questionnaire, with the specific design shown in Fig. 4.

### Statistical analysis

Statistical analysis will be performed using SPSS 27.0. Normality of continuous variables (e.g., blood pressure values, biochemical indicators) will be assessed using Shapiro-Wilk tests. Normally distributed data will be presented as mean ± standard deviation (x̄ ± s) and analyzed with independent t-tests for between-group comparisons (intervention vs. control) and paired t-tests for within-group changes (baseline vs. follow-up). Non-normally distributed data will be reported as median (interquartile range) and analyzed using Mann-Whitney U test. Categorical variables will be expressed as counts (%) and compared via the chi-square test. A two-sided p-value < 0.05 will indicate statistical significance.

Intention-to-treat analysis will be prioritized [19], with multiple imputation for missing data. Subgroup analyses will be performed for age, hypertension severity, and comorbidity status. In addition, insights on implementation barriers and improvement strategies derived from focus groups and interviews with healthcare professionals will be systematically analyzed using thematic analysis.

The economic evaluation is integrated into the quasi-experimental trial design, comparing the IoT-based hypertension surveillance system (intervention group, IG) with standard care (control group, CG). Costs and outcomes are assessed over a 12-month period to determine incremental cost-effectiveness. The analytical method is Incremental Cost-Effectiveness Ratio (ICER) [20], which is calculated using the following method. Where: ΔC = Change in mean costs (intervention vs. control). ΔE = Change in mean effectiveness (e.g., blood pressure control rate). Bootstrap simulation with 1,000 replications will be conducted to generate ICER distributions, plotted graphically on cost-effectiveness planes.

$$\:ICER=\frac{\left({\Delta\:}{C}_{IG}-{\Delta\:}{C}_{CG}\right)}{\left({\Delta\:}{E}_{IG}-{\Delta\:}{E}_{CG}\right)}$$

### Process evaluation

Besides the survey data gathered in the trial, qualitative data will also be collected to assess the intervention process. Qualitative data will be collected through questionnaire surveys with 2000 hypertension patients and focus groups with 12 healthcare providers (doctors, nurses, and administrators) to explore implementation barriers, user experiences, and perceived effectiveness.

### Ethics

The research is conducted in accordance with the ethical guidelines and the Helsinki Declaration. Ethical considerations are prioritized throughout all phases of the study, including recruitment, data collection, storage, analysis, and reporting. Participation in the research is entirely voluntary, and all participants are provided with detailed information about the study objectives, procedures, and potential risks and benefits during the recruitment phase. Written informed consent is obtained from each participant prior to enrollment, ensuring their understanding and agreement to participate in all aspects of the study. Throughout the study, participants are encouraged to contact the research team with any questions or concerns. Data collection is carried out with respect for participant privacy and confidentiality, and all reports and publications will anonymize participant identities to prevent disclosure of personal information.

The research protocol has undergone comprehensive review and obtained approval from the Ethics Committee of Beijing Luhe Hospital, Capital Medical University (Approval No. 2024-LHKY-044-02), ensuring compliance with ethical standards for clinical research in primary healthcare settings. The study protocol has been registered, and all procedures align with the ethical principles of beneficence, non-maleficence, and respect for participant autonomy. Any modifications to the study protocol will be submitted for further ethical review and approval as necessary.

## Discussion

This study represents one of the first attempts to evaluate the effectiveness of an IoT-based hypertension surveillance system in primary healthcare settings in China. The integration of real-time monitoring, automated data analysis, and dynamic performance evaluation tools addresses critical gaps in traditional hypertension management, such as fragmented data collection and low patient adherence. By combining quantitative outcomes (e.g., blood pressure control rates) with qualitative insights from patients and healthcare providers, the study offers a comprehensive understanding of the system’s feasibility and impact.

A key strength of this study lies in its pragmatic quasi-experimental design, which reflects real-world conditions by evaluating an existing intervention within routine clinical practice. The large sample size (*n* = 2000) and rigorous baseline matching enhance the generalizability of the findings to similar urban and suburban primary care settings. Additionally, the inclusion of cost-effectiveness analysis provides valuable evidence for policymakers to allocate resources efficiently.

However, several limitations should be acknowledged. First, the non-randomized allocation of participants (due to the pre-existing deployment of the IoT system in selected locations) may introduce selection bias. Although baseline matching will be used to mitigate confounding, unmeasured variables (e.g., socioeconomic status) could influence outcomes. In addition, the study’s reliance on electronic health records for control group data may lead to inconsistencies in data quality compared to the IoT system’s automated measurements.

### Implications

This study provides critical evidence on the effectiveness and implementation of an IoT-based hypertension surveillance system in primary care settings. By integrating technology with traditional healthcare delivery, the intervention demonstrates potential to significantly improve hypertension control rates and standardize care practices, particularly in resource-limited areas. For clinical practice, the real-time monitoring system enables timely interventions while empowering patients through enhanced self-management capabilities. The study’s mixed-methods approach [21] establishes a valuable framework for evaluating technology-driven health interventions, with findings that could guide wider adoption of similar systems for chronic disease management. Ultimately, this research contributes to advancing healthcare quality and operational efficiency through innovative digital solutions.

## Supplementary Information

Below is the link to the electronic supplementary material.

Supplementary Material 1

## Data Availability

Not applicable.
